# Experimental Investigation of the Effects of Machining Parameters on the Performance of Form-Cutting Tools Manufactured by Wire Electrical Discharge Machining (WEDM) and Grinding Processes

**DOI:** 10.3390/mi14101971

**Published:** 2023-10-23

**Authors:** Amir Alinaghizadeh, Mohammadjafar Hadad, Bahman Azarhoushang

**Affiliations:** 1 Department of Mechanical Engineering -Manufacturing Group, University of Tehran-Kish International Campus, Kish 79416-39982, Iran; amir.alinaghizadeh.abiazani@hfu.eu; 2 Institute of Precision Machining (KSF), Hochschule Furtwangen University, 78532 Tuttlingen, Germany; ba@ksf-hfu.de; 3 School of Mechanical Engineering, College of Engineering, University of Tehran, Tehran 14399-57131, Iran; 4 Department of Mechanical Engineering, College of Engineering and Technology, University of Doha for Science and Technology, Doha P.O. Box 24449, Qatar

**Keywords:** WEDM, form turning, machining performance, surface quality, form grinding

## Abstract

In this research, a comparison between two methods of grinding and WEDM and the chip formation of each form tool was studied through a set of designs of experiments. A multi-functional form tool with different cutting-edge shapes was designed to compare different production methods, and a grinding machine and a five-axis wire-cutting machine were made. The form tools by the wire cutting method were made with three different machining states, rough, semi-finish, and finish. The results of the experimental test showed that the chip formation of the finished surface of the wire cut tool was close to the ground tools. Additionally, the tool life in wear generation was assessed, revealing that the tool generated through the wire-cutting method with three passes exhibited superior performance compared to alternative approaches. Furthermore, employing the wire-cutting technique with high surface finishing yielded optimal outcomes for producing form-cutting tools featuring complex profiles.

## 1. Introduction

### 1.1. Form-Turning Operation and Its Applications

The form-turning process using a form tool is one of the methods used to create a form on parts. Although this method cannot be used for parts with a small diameter, e.g., in MEMS applications, due to the high contact surface of the cutting tool with the workpiece and much machining, this method is always a suitable alternative for the “point-to-point turning process with interpolation” and using a cutting tool with a point contact [1]. In this reverse method, the desired form is created on the tool and is carried out with radial, axial, or angular machining movements. Like many cutting tools, these are made from available materials [2,3]. The choice of the material of the form tool is directly related to the material of the workpiece and machines, how to apply the load, the cutting depth, the cutting speed, etc. In terms of tooling methods, these tools are integrated and replaced in different ways. Different types of form tools can be used in different machining situations. The cutting speed of these tools depends on their working conditions, including the type of machine and the material of the workpiece. Single-edged tools used in processes such as turning and planning or multi-edged tools used in processes such as milling or drilling can be considered subsets of form tools [4].

### 1.2. Production Techniques for Form Cutting Tools: A Comparative Analysis

Three methods are used to produce form tools [5,6,7]. In order to produce a large number of forming tools, the tungsten carbide tools can be a good option that can be produced by the powder metallurgy method. In this method, with proper molding, it is possible to produce tools with any desired shape several times. The method that is used to make form inserts with a lower production rate is the grinding method. This method forms a raw insert made by the powder metallurgy method into the desired shape using sharpening grinding machines. The inserts are ground in two ways to make the form insert [8]. In the first case, standard grinding wheels available on grinding sharpening machines, such as flat, plate, bowl grinding wheels, etc., are used for machining the form areas. Each area of the form is made into the desired shape with one of the standard grinding wheels, and finally, using several grinding wheels creates the final shape of the form tool. The following method is profile grinding, which has the same profile as a single grinding wheel [9]. The difference is that special abrasive materials such as CBNs must be used for grinding cutting tools, which have very high mechanical strength. Secondly, the production number of form tools is much lower compared to the production number of workpieces. The third form tool production method uses electric discharge with a wire-cutting machine. The special operation of making form inserts using a wire-cutting machine allows the required elements to be created on the tool with high speed and accuracy. The form tools produced by wire-cutting or machines must have the characteristics approved by the customer, such as the desired surface quality and correct geometry, after the final finishing stage. Although the EDM method (wire-cut) is always considered an alternative or sometimes the only available method, it is a severe competitor to grinding. However, to achieve this goal, obtain tolerances, and pay the desired level in form tools, they usually use the grinding process as the final process. The reason for this is the method’s high speed and efficiency [10,11,12]. A form tool with a cutting edge has the reverse shape of the workpiece to be chipped. The form tool can be used in a single-step operation in terms of the number of movements. Various motions, including forward or backward radial or axial movements, are created for it using different axes of CNC machines. These tools can act as a machine, by applying advanced movement and infeed in the workpiece of different diameters. Before using the form tool, the outer diameter of the workpiece is machined using other tools. Usually, after performing the machining using the form tool, a significant part of the workpiece is prepared [13]. According to the profile created on the grinding wheel, there are challenges in producing form tools using the grinding process. The development of CAD/CAM methods makes it possible to create unique profiles using point diamond tools on grinding wheels (using point heads in numerical control programs). However, the specific shapes of grinding wheels have yet to be studied [14]. Moreover, regardless of how a form tool is created, the general principles of chip formation will be the same. Due to effects such as the thermal effect on the cutting edge obtained by electric discharge or the residual stresses caused by the machining forces in the grinding process, there may be minor effects on the way the chips are formed. However, it is always possible to use a general behavior [15]. Special methods in manufacturing tools based on electric discharge machines allow continuous production when the process is quickly and correctly carried out. To create a suitable analysis for such tools, the manufacturers of cutting tools use the grinding method and the electric discharge method to polish the cutting edge of the tools. Choosing one of these two methods depends on characteristics such as geometry and the quality of the final surface of the cutting edge. In order to achieve the tolerances and the quality of cutting edges needed in cutting tools, manufacturers usually use the grinding method because it is carried out in a series and has good productivity [16]. However, the electric discharge method with wire cutting can be a better choice and is sometimes the only possible choice. Most research has been conducted to improve the accuracy during the WEDM machining process, especially in creating cutting contours. The main limitation of the WEDM process is the relatively low machining speed compared to other non-traditional machining processes, such as laser cutting or thermal cutting. Finally, most research has advanced the WEDM process in terms of improving productivity, increasing accuracy, and reducing vibration or wire deflection [17]. The growth and development of the machining process have caused the need to produce form tools with high hardness and complex shapes to be considered. Using the wire electrical discharge process to produce form tools is a method that can achieve this because of its unique features [18]. The input parameters of the electric discharge process with wire are: pulse on time, pulse off time, peak current, material removal rate, servo voltage, gap, narrow gap, gap voltage, guides, and wire tension.

### 1.3. Comprehensive Investigations of Various Production Methods for Form Tools: Performance, Characteristics, and Comparative Analysis

Several kinds of research have been carried out to evaluate the different aspects of the forming process with tools made using different production methods. Tadeusz Mikolajczyk et al. [19] selected a carbide cementite tool for their tests, and the tool was made by wire cutting and grinding methods. The tool life and manufacturing time of the tools and the roughness of the machined part using these two tools have been investigated and compared. According to this research, the lifetime of tools produced by the wire-cutting method is almost half the lifetime of tools produced using the grinding method. Furthermore, the roughness of the machined surface with the tool produced using the grinding method is less than the roughness of the machined surface with the tool produced using the wire-cutting method. On the other hand, the production time of the wire-cutting form tool is less compared to the production time of the grinding form tool. Nagabhushana et al. [20] investigated the differences and inherent similarities between the two processes electro discharge sawing (EDS) and wire-electro-discharge machining (WEDM). In this research, HSS and titanium were used for the test. One of the notable observations was the higher erosion rate of titanium compared to HSS in both processes, which is explained by the reduction of energy concentration in HSS due to its high thermal and electrical conductivity. Furthermore, the surface roughness obtained in EDS is higher than in WEDM. Mamalis et al. [21] compared the production process of form tools from three methods of grinding, electric discharge with wire, and the two-stage process of electric discharge with wire, in addition to using a rotary wheel tool as an EDM broaching electrode, which was discussed. Kashif Ishfaq et al. [22] investigated the parameters of the pulse on and off time, voltage, wire tension, and dielectric pressure in an electrical discharge with wire to produce form cutting tools. Huang and Kuo [23] studied the multi-step machining of HSS made by powder metallurgy using the WEDM process. Klocke et al. [24] conducted research by changing the cutting angles in the form tool, such as the chip, wedge, and flank angle, during the chip removal process. The goal was to obtain the geometric correctness of the form contour in the cutting tool. The cut in this research was carried out in five stages (passes). The first stage’s goal was maximum chipping and rough machining with maximum energy and minimum wire tension. The second and third machining stages were semi-finishing and finishing to achieve precise geometric dimensions. The fourth and fifth stages were the stages of achieving the maximum quality of the surface and the extra finish mode, which was achieved by changing the polarity of the wire and the workpiece. The research was carried out using the broaching process, and its goal was to obtain the optimal working angle through calculations. Furthermore, the working angle was defined according to cutting and advancing speeds. Calculating the optimal working angle makes it possible to estimate the amount of tool wear. In another study, Klocke et al. [25] calculated the chip geometry and machining forces in the process of hub turning with a hub tool with shaped cutting edges. This research aimed to obtain the highest efficiency and optimal machining conditions based on the shape of the cutting edge of the broaching tool. Finally, the obtained machining forces were compared with the forces applied to the machine spindle during the test. The tool wear and the machining surface quality were also compared with the test work based on the shape of the chips. Tapoglou et al. [26], using the broaching method, evaluated the machining forces and wear of form tools used in producing helical and simple gears. The goal was to control the final price and quality of the manufactured gears. The software resulting from coding the movement of the tool on the workpiece was created with accuracy and the ability to calculate movements from different angles, which has provided the ability to simulate the process in conventional design CAD software. In another paper, Tapoglou [27] researched the form inserts mounted on the tool holder used in the skiving method of external and internal gears. This research presented a method for simulating this process in predicting undeformed chips. With the extracted codes, it was possible to simulate in CAD software. Bouzakis et al. [28] studied chip formation in the broaching process with tools with an involute form of gear teeth. The mathematical analysis performed was based on the calculations of the way the form tool and the groove between the two gear teeth engage. This research resulted from modeling and coding using the finite element method. One mode for simple gear machining and four possible modes for spiral gear machining were considered [14,29]. The grinding results were compared with the test work, and finally, the contact of the chip with the side surface of the form tool was investigated.

### 1.4. Optimizing Form Cutting Tool Manufacturing through a Comprehensive Study on Electrical Discharge and Grinding Methods in the Current Study

This research has sought to find the optimal mode in manufacturing form tools with the highest speed and accuracy. First, to obtain a model for the formation of chips in form tools, and using preliminary tests, the behavior of chips during the turning process with form tools was studied to perform the analysis steps of the process based on it. For this purpose, the desired form was created by two methods of electrical discharge with wire and profile grinding on HSS tools. Three states of roughing, semi-finished and finished, were considered in the form tools throughout the electrical discharge process. First, the characteristics of the free surfaces of the form tool produced by the two mentioned processes before and after turning the form were investigated. Then, the forming process was carried out through three machining conditions: rough, semi-finished, and finished, and the characteristics of the machined workpiece, including roughness, texture, and surface characteristics, were examined. The material used for the workpiece was also chosen due to its wide application in the industry, availability, and the required mechanical parameters to cause wear on the tool (in the small number of machining steps).

## 2. Research Methodology

**Figure 1:**
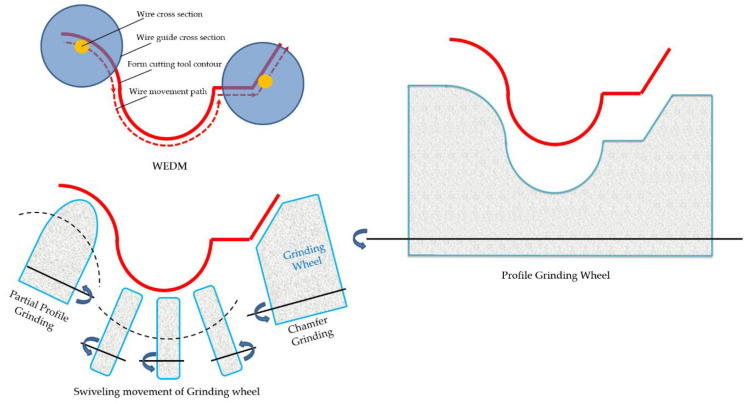
A schematic view of the manufacturing methods of the form tools used in this study.

As previously mentioned, several methods are used to make form tools. Figure 1 shows the manufacturing methods for the form tools used in this study in a schematic view. The experimental test was designed to examine the differences between the output parameters of form turning in the case where the tool is made in different ways. The final characteristics of the cutting tool and the workpiece, which are affected by the manufacturing method, are finally measured.

High speed steel (HSS) as the cutting tool material was used to investigate the effect of the different manufacturing processes on the form tool characteristics. The chemical composition and material properties of the utilized HSS are shown in Table 1.

The Design of Experiments (DOE) was prepared based on the use of different cutting tools that were made using different methods. The following three methods made form tools made with wire cuts. One pass of wire movement to create a form profile on the cutting edge of the tool (roughing with wire-cutting), two passes of wire movement (semi-finished work with wire-cutting), and three passes of wire movement (finishing). One of the tools was made using the profile grinding method, and the rest were made using the electric discharge method with a wire-cutting machine.

The production of form tools using the WEDM process was created using a Mitsubishi MX600 (Mitsubishi company German Branch, Ratingen, Germany), a universal wire-cutting electrical discharge machining tool. According to previous studies, a self-developed closed-loop constant tension control system was used to control the electrode wire tension to improve the machining stability. In the next stage of the form-turning process, the parameters of the lath were determined so that the three machining conditions of rough, semi-finished, and finished machining were realized. Table 2 shows the machining conditions according to the selected parameters. After reviewing the values introduced in relevant articles in this field, the cutting parameters were selected based on experimental work within various ranges of cutting speed. The maximum cutting speed was chosen to accommodate the form-turning operation considering the material of the workpiece. The minimum cutting speed was also selected in a way that ensured the economic production of the workpiece was not compromised.

**Figure 2:**
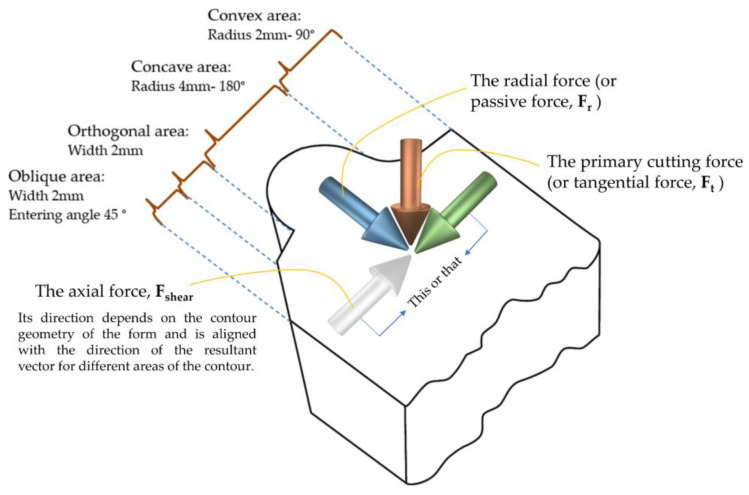
The contour of the selected form includes all four forming shapes.

As shown in Figure 2, the contour of the selected form, which includes all four characteristics for each type of profile form, is based on the combination of four modes, i.e., concave curvature, convex curvature, flat surface (orthogonal area), and oblique surface. Purposefully, the symmetry of the contour concerning the axis of the tool was not taken into account in order to be able to make maximum use of the different areas of the contour of the form (due to the variety of characteristics on it) during the formation of the chip and its flow.

**Figure 3:**
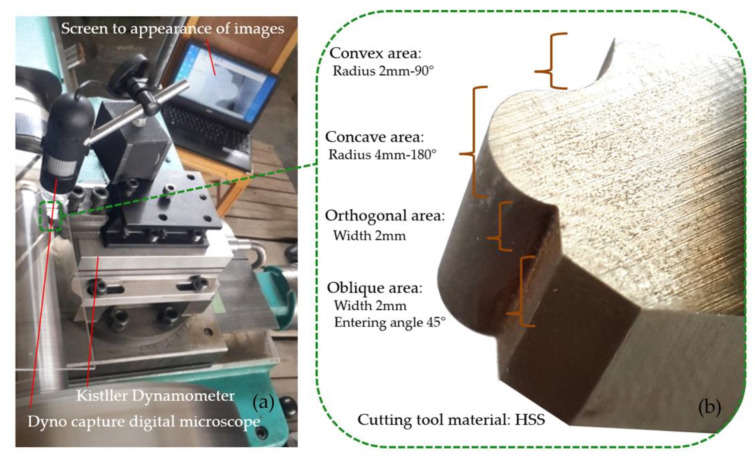
(**a**) The form tool, tool holder, dynamometer, and portable digital microscope on the turning machine; (**b**) the fabricated form tool using the profile grinding process.

To set up the experimental test, the HSS form tool was installed in the tool holder, and the holder was attached to the dynamometer. Figure 3a presents the turning machine’s form tool, tool holder, dynamometer, and portable digital microscope. Considering that one of the goals of this research was to investigate the wear of form tools produced in different ways, the workpiece’s material was chosen so that it was possible to create wear in the tool in the designed tests. On the other hand, it has many industrial applications and a reasonable price. Thus, AISI 4340 steel (Iran Alloy Steel Co., Yazd, Iran) was selected due to having an acceptable level of initial hardness. In the sense that, even prior to secondary operations such as hardening, for machining experimental tests, hardness resulting from steel production operations is, to an extent, where one can view the occurrence of wear on the cutting edge of the cutting tool. Its characteristics include heat treatment and resistance to heat and use in parts that are exposed to tensile, bending, and torsional loads. Examples of its applications include long machine parts that require high tensile strength and impact resistance, such as crankshafts, disk plates, eccentric shafts, valve shafts, and toothed parts. Figure 3b shows the fabricated form tool using the profile grinding process and addresses the combination of various form contour geometries in a single form tool.

**Figure 4:**
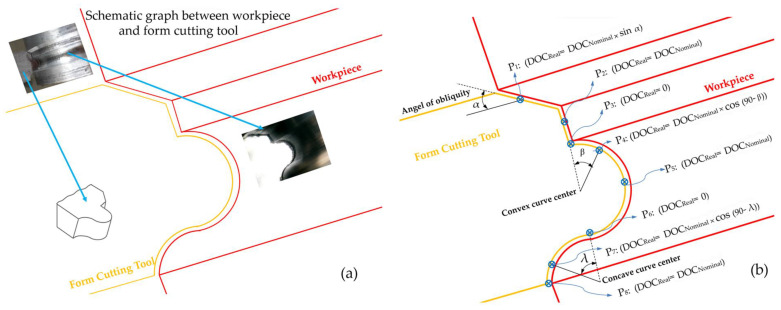
(**a**) Schematic of the contour of the form and the position of the engagement of the cutting tool and the workpiece, (**b**) The actual size of the cutting depth in different areas of form contour.

In this research, according to the dimensions of the selected cutting tool, the diameter of the workpiece was considered to be 40 mm and its length was determined to be 300 mm, as part of it was placed in a three-jaw chuck of the turning machine and part of it was in contact with the tail stoke, so that it could be adjusted in line with its length. The cutting forces were recorded using a Kistler dynamometer, and the data were saved on a computer. After filtering the noise, the raw data could be used for data analysis based on the machining time. To examine the chips and the process of their formation, a schematic of the contour of the form and the position of the engagement of the cutting tool and the workpiece is shown in Figure 4. In Figure 4a, the cross-section of the workpiece after entering the tool and the contour of the form created on the cutting tool is visible. As indicated in Figure 4b, there are different cutting depth values at every point of the form contour. A nominal size can be considered for the depth of cut, and in the turning process, this value is dependent on the amount of feed per round (f_n_). On the other hand, according to the entry (approach) angles of different points of the formed contour, it is possible to obtain the actual size of the cutting depth per one revolution of the workpiece for every point.

The relationships related to the four characteristics introduced for the variation of form contours at each position are shown in Figure 4b. On surfaces with convex and concave curvature, the actual size of the cutting depth can be obtained according to the position of each point of the circle sector and the angle of the radius drawn from the center of the arc to the desired point with the workpiece axis. On a flat surface, because the entry angle (the angle between the surface and the workpiece axis) is zero, the actual and nominal cutting depths are equal. On the oblique surface, the size of the actual cutting depth depends on the angle of inclination (diagonality) of the contour surface to the axis of the workpiece.

## 3. Results and Discussion

### 3.1. Cutting Forces Evaluation

**Figure 5:**
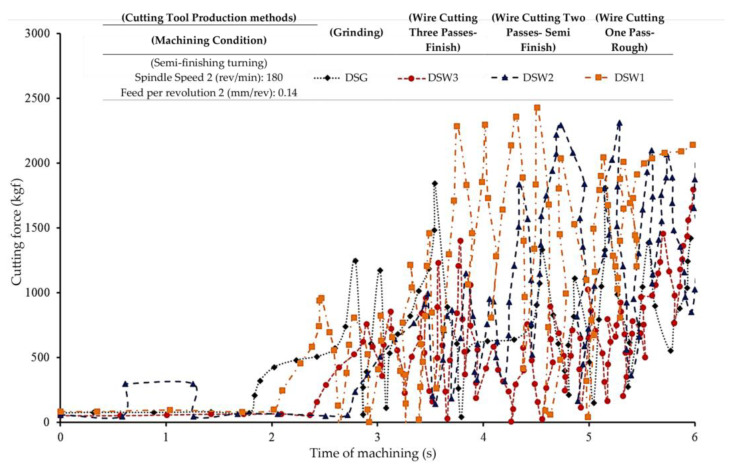
Cutting forces of the form-turning process obtained using the dynamometer.

In the machining tests, the amount of overhang of the used form tools was the same, so the value of cutting forces was related to the contact cross-section of the form tool and the workpiece and the cutting-edge slowness along the contour of the form. By increasing the cutting depth from the frontmost point of the formed contour to the rearmost point, contact with the workpiece was achieved, and obviously, the cutting forces gradually increased. According to Figure 5, the pattern of force changes due to the involvement of different areas of the cutting edge of the form tools was the same. The correct comparison of the magnitude of each component of the cutting force between the tools made by different methods was correct after completing the cutting depth when all the areas on the contour of the form were in contact with the workpiece. In addition, by observing the fluctuations (noise) of each tool’s cutting forces, the amount of vibration during the machining of the form operation could be understood. In all the tools used in this research, by increasing the cutting depth and subsequently increasing the contact surface of the tool and the workpiece, the fluctuations (noise) of the graph of the components of the cutting forces increased, which indicated a gradual increase in vibration. By averaging the graphs obtained, it was observed that the peaks and valleys of the force graph were somewhat the same for all the tools used.

In form tools, due to the distance between the front and back (rear) points of the contour, the contact surface between the tool and the workpiece will gradually be completed over the machining process. This is because form tools have different orientations in the cutting edges in different areas of the form contour. During the gradual contact of each of these regions with the workpiece, there will be a mutual interaction between the systems of cutting forces. Therefore, until the cutting depth is completed, the forces have an increasing trend depending on the contour profile of the form. From then on, their value is proportional to the length of the cutting edge involved in the tool, which is constant in the radial and tangential components and variable in the axial component depending on the symmetry or asymmetry of the contour of the form. The results of the dynamometer comparison of four different production methods of form tools in terms of mechanical forces are presented in Table 3.

In the present research, since the profile of the selected form is the same, it is possible to consider the behavioral trend for the recorded cutting forces. In the diagram of the cutting forces of all of the tools, it was observed that before the tool ultimately enters the workpiece and the engagement level between the tool and the workpiece is completed, sometimes the force magnitude is greater than the force value after full engagement. The reason for this is the lack of stability in the balance between the cutting forces generated in different areas of the form contour, which are located with different orientations regarding the direction of the infeed of the tool. After that, with similar behaviors observed between the tools made through different methods, a decrease in force magnitude and an increase in fluctuations (noise) are seen due to the existence of a maximum contact surface between the form tool and the workpiece.

Due to the uniformity of the form contour profile in all of the used form tools, the difference in the magnitude of the machining forces can be related to the sharpness of the cutting edge. According to the recorded forces, the cutting force diagram of the form tool made through grinding has fluctuations (noise), which also corresponds to the surface of the workpiece created from it. In terms of the magnitude of the cutting forces, the form tool created using the electric discharge method with one pass has the highest value, which indicates the absence of sharp cutting edges along the contour of the form. At the beginning of cutting each area of the cutting edge, the recast layer immediately collapses, and the chipping operation is performed, with the cutting edge leaving the remaining residue. Therefore, a surface with low surface quality (corresponding to the recorded images of the machined surface of the workpiece) is prominent. In the form tool created by the two-pass WEDM process, the cutting forces, along with their fluctuations, have a peak point, but after that, a significant decrease in both occurs due to the loss of the recast layer on the flank surface of the cutting edges. The lowest number of cutting forces can be seen in the tool made using the WEDM process with three cutting passes. It can be expected with this tool due to the reduction of the recast layer, and because there is the highest sharpness of the cutting edge, which is a factor in reducing the fluctuations (noise) of the cutting forces. The recast layers in this manufacturing method were so insignificant that, after the cutting edge came into contact with the workpiece, their removal had the minimum effect on the sharpness of the cutting edge, and chipping was performed under suitable conditions.

### 3.2. Surface and Texture Analysis

**Figure 6:**
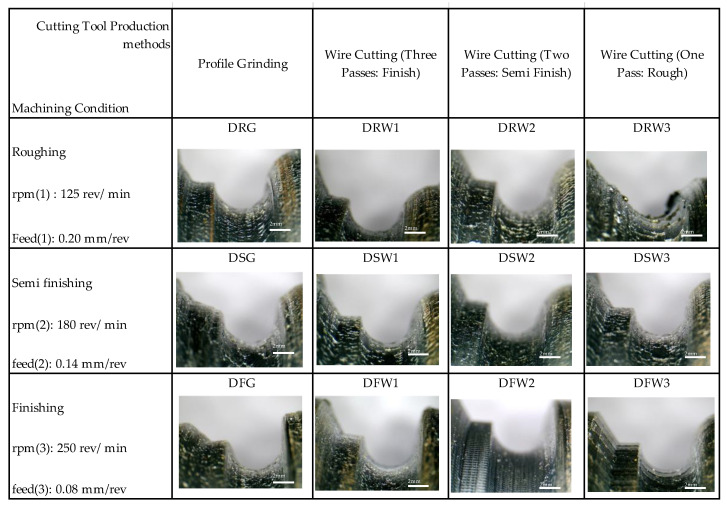
The variation of workpiece surfaces after machining under different cutting conditions.

The variety of workpiece surfaces after machining under different cutting conditions is shown in Figure 6. The presence of lines resulting from tool vibration or the remaining particles separated from the surface of the form tool can be seen. In this figure, the cutting parameters used during machining were neglected only to show the differences in the obtained data. Each level aligns with a specific machining parameter setting outlined in Table 3. The differences can be seen in this form, the presence of the vibration of the tool, the presence of burs on the surface, and the joining of BUEs separated from it. The design of the machining test concerning the method of making the tools was entirely correct, and the desired quality level in the workpiece can be a criterion for choosing the method of making the form tools.

**Figure 7:**
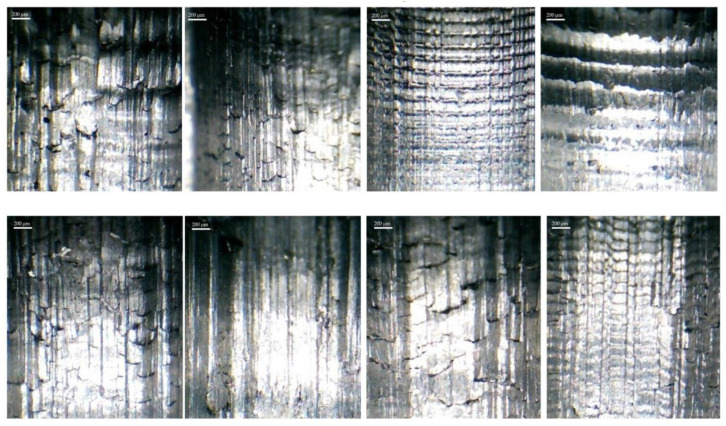
The surface texture of the different samples based on cutting tools.

According to the test design introduced in Table 2, the tests pertaining to the dry machining mode were performed, and the data obtained from the surface texture of the samples are compared in Figure 7. In this test, the effect of the parameters of the cutting speed, the feed rate, and the cutting tool manufacturing method was investigated on the texture of the workpiece surface. The effects of vibration can be seen in the surface finishing for tools made with one and two wire-cutting passes. This vibration is increased to some extent until the chattering phenomenon is observed on the surface of the parts. Considering this phenomenon’s inappropriate effect on the workpiece’s surface quality, dimensional accuracy, and the tool and machine life, understanding it can help improve the economic aspect of machining. Its occurrence can be prevented through the optimal selection of chipping parameters.

**Figure 8:**
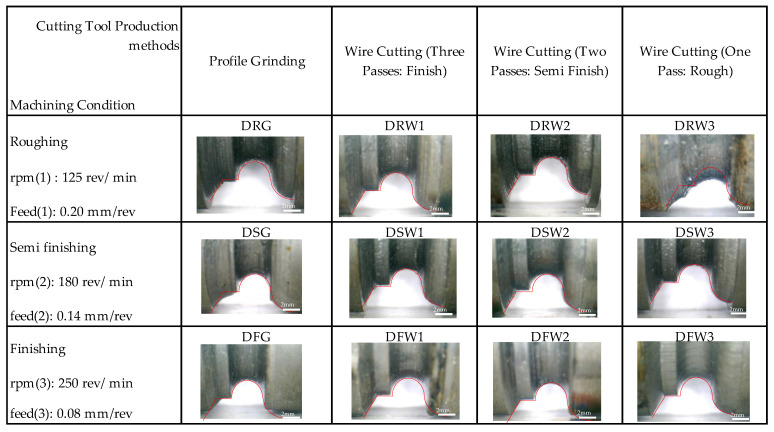
The contoured form of different samples with various machining conditions.

In roughing mode machining and for the tool made with a wire-cut cutting, it was observed that there were burrs on the surface of the workpiece. Furthermore, in terms of the geometric accuracy of the form contour, this mode was the weakest. It can be seen in Figure 8 that, after matching the form contour, the lowest matching is related to this mode. This mismatch may be due to the severe wear on the tool’s cutting edge along the contour of the form. Figure 8 shows the matching of the initial form contour file to the image of the workpiece with higher magnification for tools made by one, two, and three wire-cutting passes and the grinding process.

**Figure 9:**
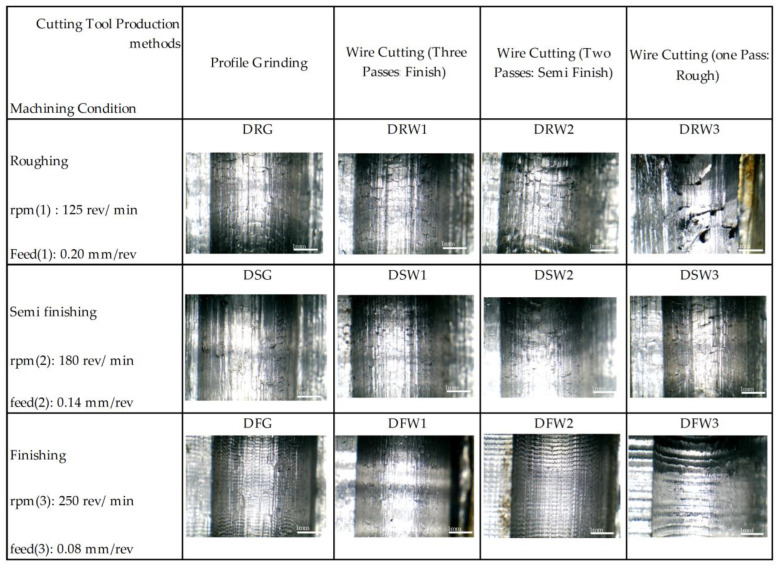
The effect of the separation of material masses and material rupture over the machining process.

More surface details can be seen by taking a picture with a greater magnification of the surface of the upper row parts of Figure 8. The details include severe plowing, galling wear, scratch (scratch) marks on the surface, the effect of the separation of material masses, and the creation of a rupture in it due to the low cutting speed, which is shown in Figure 9. As this figure is precise, the surface of the workpiece is in a more suitable condition in cases where the tool is made by three-pass grinding and wire-cutting methods. Except for the texture of the surface and studying its details, comparing the roughness is also very helpful in this regard. In this case, due to the limitation provided by the form’s contour, it was impossible to measure the roughness along the axis of the workpiece.

### 3.3. Chip Formation Study

**Figure 10:**
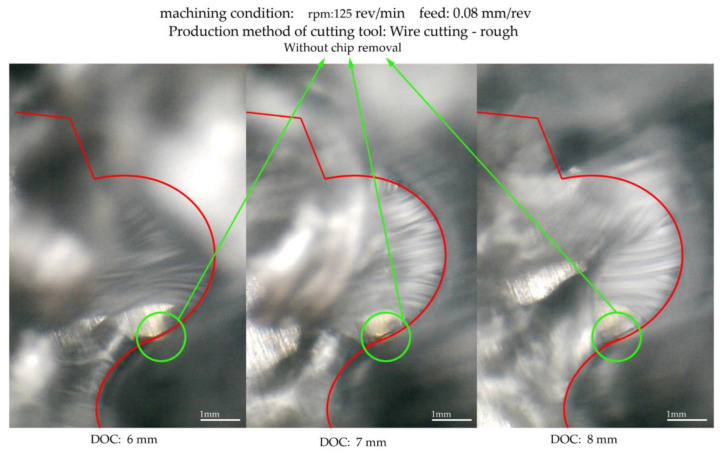
Chip formation on the rake surface of a form tool that was made via wire cutting with a rough machining condition.

**Figure 11:**
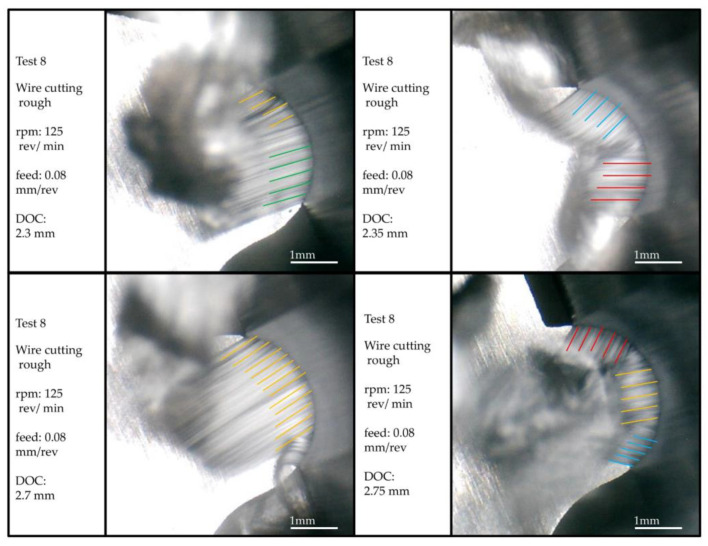
The chip flow directions (colored lines) and their continuity observation.

The second purpose of the machining test was to study the chip formation process. For this case, two measurement methods were used. Figure 10 and Figure 11 display measurements taken through online measurement. Specific points in the contour of the form are essential because they may differently appear in terms of the wear behavior and impact on the chip formation. Chip formation on the rake surface of a form tool that was made via wire cutting with one cutting pass, with a rough machining condition, is shown in Figure 10. According to this figure, the considered form contour’s inflection point, the surface’s connection (junction) with convex and concave curvature, appears without chip removal. This contour area has the same behavior in the phased images recorded at different cutting depths.

Observing the local (regional) chips formed in the two areas adjacent to the inflection point, it is clear that their flow directions are entirely different. The direction of the chips formed on the convex curve was toward the center of the convex curve, and the direction of the chips formed on the concave curve was toward the center of the concave curvature. In addition, it can be observed that, at this point, the infeed direction of the tool into the workpiece is parallel to the direction of the cutting edge. According to the information provided in Figure 11, the inflection point is located in the area with the lowest actual cut depth. Therefore, from the above statements, it can be concluded that the thickness of the undeformed chip around this point is so low that, first, its flow direction is affected by the neighboring chips. Second, due to the tool’s vibration during chip removal, before reaching the chip formation region’s inflection point, the material in that part is removed by points adjacent to the inflection point on the cutting edge closer to the axis of the workpiece. In this case, the inflection point does not have a chip.

Another critical point that can be observed during chip removal is that the chip flow directions and their continuity and discontinuity in different areas of the form contour are constantly changing at different cutting depths. This means that the directions of the chip flow follow a general principle, and that is the alignment with the normal vector of the contour shape. However, they repeatedly break from points, and after a slight change in the depth of the cut, continuity in their flow is again observed. Therefore, apart from the constant changes in the cutting speed at different points of the form contour, there is another factor in these breaks and connections. This matter is discussed below.

**Figure 12:**
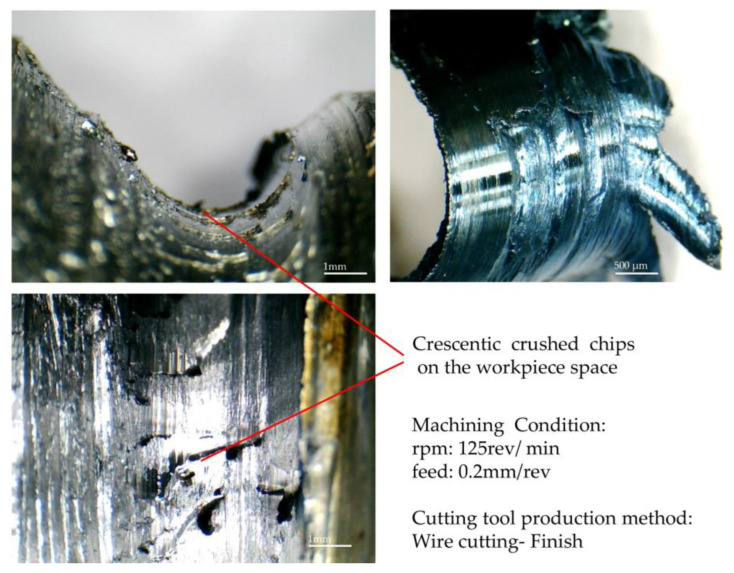
Chip formation study of convex form turning.

Another point that can be inferred from Figure 11 is that the direction of the chips’ flow in the area where the chips continuously appear is the result of the transverse contour normal vector from the onset to the end of the continuity area of the chip. In other words, the influence of the neighboring points necessitates that the initial assumption regarding the chip flow aligned with the normal vector of the contour at each point be modified. In this way, the continuity of the chip and the angle of the normal vector from the beginning to the end of that area is effective in the direction of the chip flow, and the final direction is a result of the variation of the normal vector angle in this range of continuity. Therefore, it can be concluded that whenever a new rupture or continuity is created in the chips, due to the change in the angle of the normal vector, the direction of continuity of the chip flow will change. Sticky (adhesive) burrs seen on the workpiece’s surface due to being in the convexly curved area of the tool can be seen in Figure 12, along with the chip formed in that area. These burrs are seen as concave in this form due to the inverse state between the form-cutting tool and the workpiece. The images were recorded in a different orientation for a better surface view. The related machining parameters were the roughing among the machining conditions considered in the design of the experiments and the tool made through a one-pass wire cut. Like the surface of the workpiece, crescentic crushed burrs are also visible on the back of the chip. One side of the chip shown in the picture has a state of saw tooth, precisely the side where the thickness of the chip was minimal. The lines created perpendicular to the axis of the workpiece can be seen on its surface, created by the rotation of the workpiece and the contact with the cutting edge of the form tool. Similar lines can be seen on the back surface of the chip.

**Figure 13:**
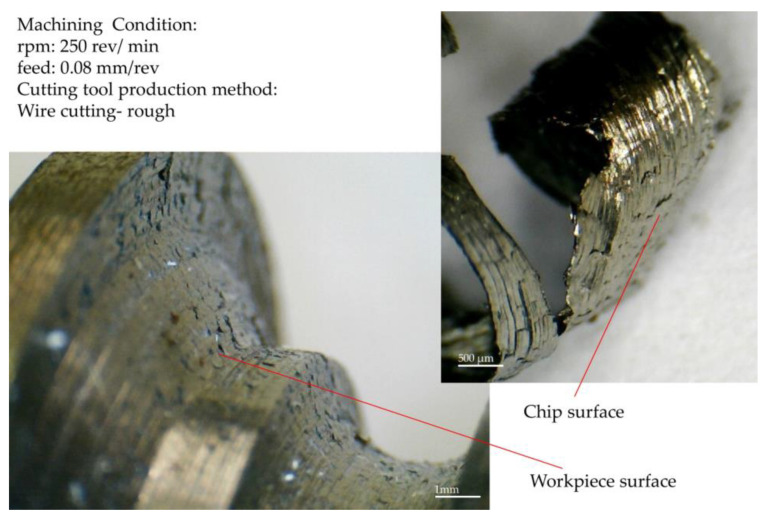
Chip formation of part with tools made with one-pass wire cutting.

Many images were recorded to prove the match between the back of the chip’s surface and the workpiece’s surface, another of which is shown in Figure 13 for finishing and tools made with one-pass wire cutting. Due to the direction of the workpiece’s rotation and the chip’s exit, tiny masses of material can be seen in this figure, the beginning and end of which are evident from the surface of the workpiece. These lumps are the effects of the stagnation area of machining exactly where the chip separates from the workpiece, which is created based on the infeed of the tool’s cutting edge to the workpiece. In some situations, built-up edges may show up as separated and joined accumulations, respectively, from the cutting tool rake face and to the chip flow, or it is possible to see them as residue on the machined surface.

**Figure 14:**
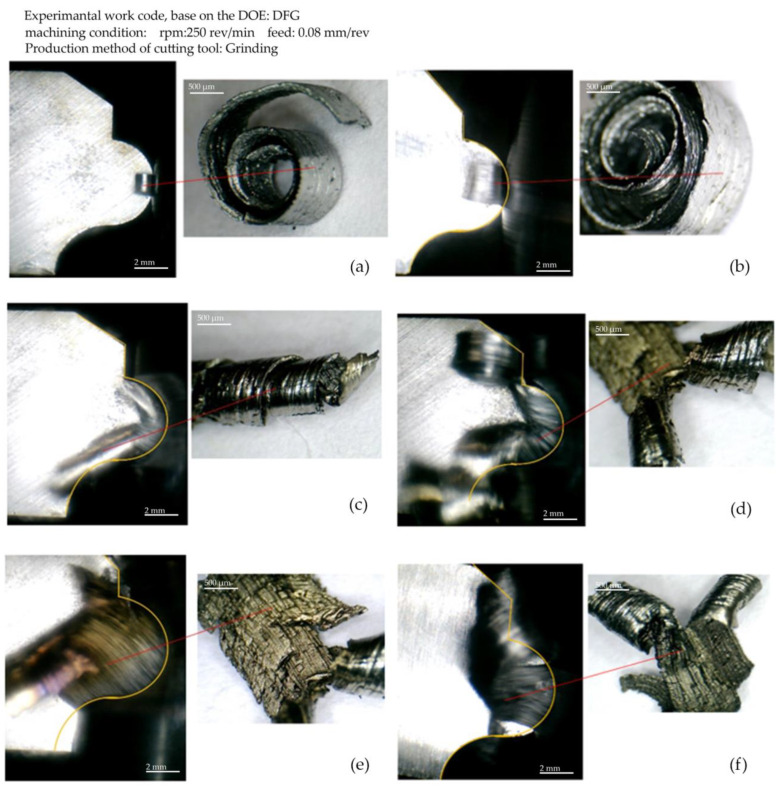
Incremental imaging of the form-turning process with tools made using the grinding method.

Figure 14 displays the chip corresponding to each area of the form contour, along with the image of that area during machining and chip formation. The images are created based on step-by-step imaging of the form-turning process with tools made using the grinding method. The machining state of the finishing was used to set the parameters. In Figure 14a, the primary chip is seen at the front position in the contour of the form on the surface with a convex curve. The chip formed in one piece, without breaking at a shallow cutting depth, is seen in Figure 14b, and Figure 14c shows the twist of the chip and its direction at a greater depth of cut after it ruptures on the rake surface of the form tool. A chip resulting from the infeed of the convex, curved surface of the tool can be seen in Figure 14d, where the moment of rupture (separation) and conversion (turning) of it into two separate chips is seen. In Figure 14e, the engagement of the surface with a concave curvature at higher cutting depths and its effect on the chip flow direction is shown. Finally, the re-joining of the chips and the forming of the new chip in one piece by the separation of the build-up edge as a rupture factor is visible in Figure 14f.

**Figure 15:**
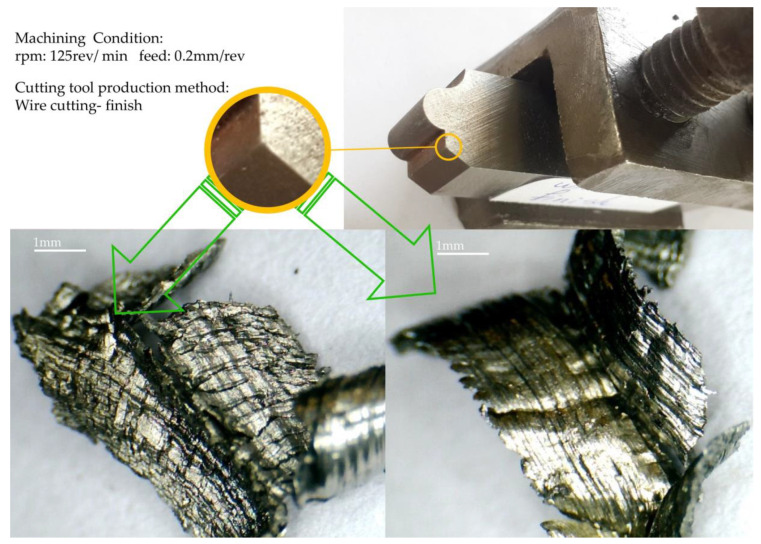
Chip formation of the junction of the flat and diagonal surfaces of the formed contour.

The effect of the cutting contour geometry on the chips is evident in the chips that continuously flowed. Figure 15 is related to the chip formed at the junction of the formed contour’s flat and oblique (inclined) surfaces. This point is considered a place to change the direction of the chip flow and its thickness. As can be seen in the figure, the front and back surface of the chip has a folded line. Actually, in some areas of the contours of the form, where the angle difference of the normal vectors is partial, the continuity of the chip may be obtained, and a flow parallel to the average of the angles of the normal vectors can be seen. The area shown in Figure 15 is one of these areas.

**Figure 16:**
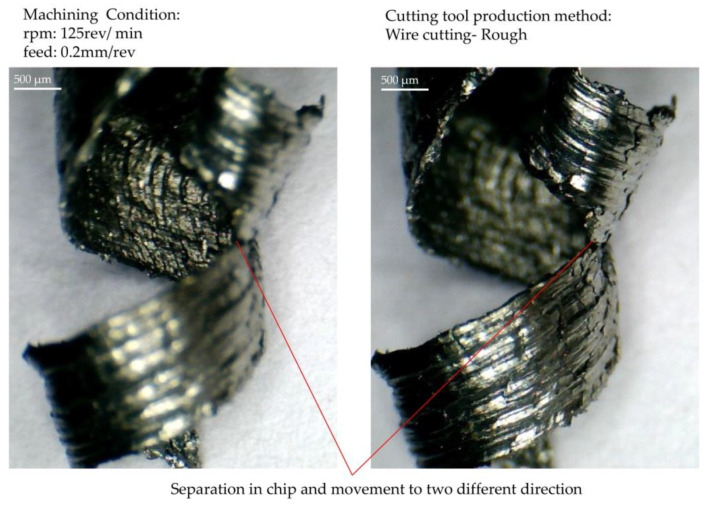
The front and back sides of the chip surface.

Chip rupturing and its flow in two different directions are depicted in Figure 16. The displayed chip is the moment of conversion (turning) the continuity of the chip into its rupture. Image clarity and sharpness have been adjusted as the near-far composition for the front and back surfaces of the chip. As mentioned earlier, the chip’s rupture and continuity are repeated at different infeed depths. Therefore, it is essential to find the cause of the rupture.

When looking at the chip shown in Figure 16 from another angle and more clearly, it is clear that, on the surface of the chip, there are differences between flow patterns, which make the chip prone to breakage at specific points. The tendency to break, as mentioned above, is directly related to the shape contour geometry and the thickness of the undeformed chip. The shape of the chip depends on the direction of the flow, the geometry of the cutting edge, and the discontinuity factor. A chip always has the necessary preparation for rupture, and the second factor that current research has always been looking for, causes it to rupture. This factor was identified in other planned tests and will be introduced.

### 3.4. Effect of Wear and Damage on the Flank Surface

**Figure 17:**
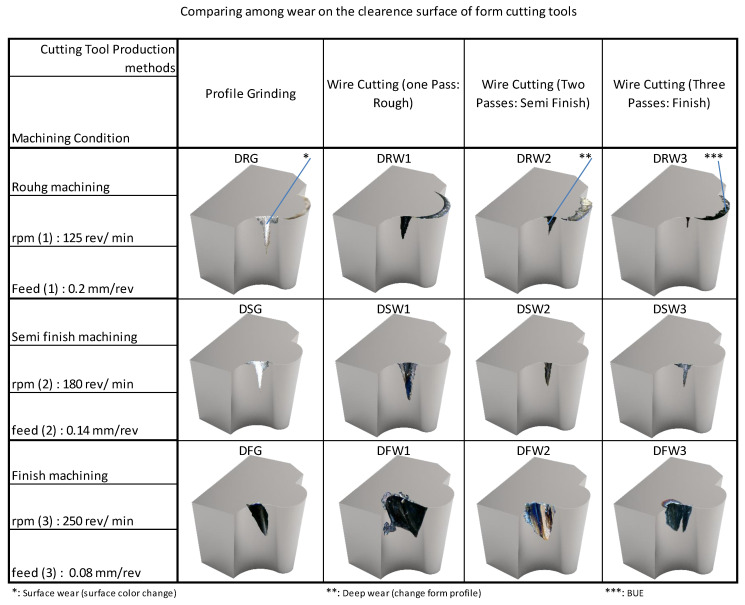
Wear and damage on the free surface of form-cutting tools.

The comparison between the wear and damage on the flank surface of form-cutting tools (under machining conditions and using different methods for their production) is given in Figure 17. The bottom row is related to the finished machining state and shows that the tools have more wear and damage than the other rows. The meaning of the state of finish machining in this research is not that by using another form tool (with rough cutting parameters) the machining is carried out until it is close to the final depth of the cut, and by changing the form tool and using the finishing cutting parameters, the remaining machining stock is removed. Rather, machining is started by a form tool and with the adjustment cutting parameters of the finish (which includes the highest level of spindle rotational speed and the lowest level of feed per revolution) and continues until the desired cutting depth is reached.

In the top row of the figure, the wear created at the inflection point of the contour of the form can be seen in the state of rough machining. In this situation, the lowest level of spindle rotation speed and the highest level of feed per revolution has been established. The results show less wear in the top row compared to the middle and bottom rows. However, according to the range of cutting speed, the built-up edge formation is seen on the rake surface of the tools. In vertical comparison, the columns are related to the manufacturing method of form tools. According to the figure, it can be seen that the lowest amount of wear was related to the tool that was manufactured with two methods of grinding and wire electric discharge machining with three passes. However, the bound displayed on the tools alone does not determine which tool is most affected during machining. For this reason, two colors were used to distinguish surface wear and deep wear, which usually lead to failure if they increase. According to the footnotes of the figure, it can be seen that although the extent of surface wear was more significant in the tools made through grinding, the depth of wear was greater in the tools made through the electric discharge method with three passes.

### 3.5. Effect of Manufacturing Methods on the Precision of Form-Cutting Tools

**Figure 18:**
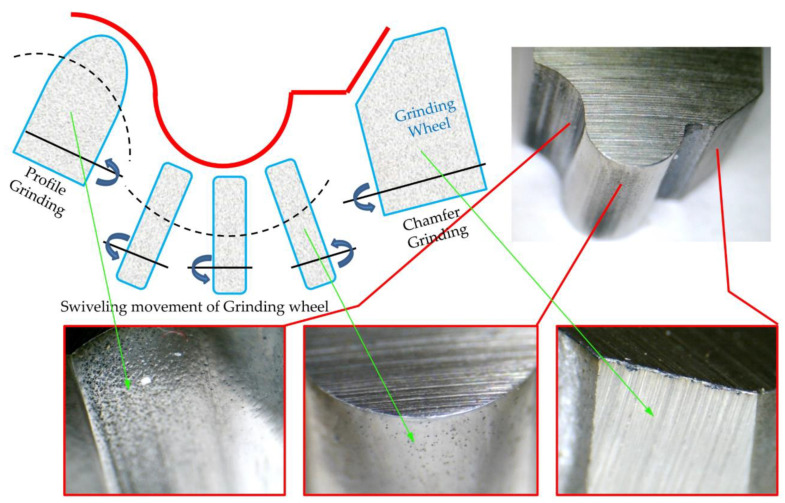
The manufacturing precision of form tools made by grinding method.

In this study, one of the goals was to find the differences in the precision of the manufacturing of form tools manufactured using methods of grinding and wire electrical discharge machining. In the planned machining tests, three situations were introduced to the tool-making using the electrical discharge method with a wire-cutting machine, which consisted of one, two, and three cutting passes, which were introduced as rough, semi-finished, and finished methods, respectively. The data obtained from the test were compared and analyzed, as shown in Figure 18, in different areas of the flank surface of the form tool made using the grinding method; a variety of quality and texture pertaining to the surface can be seen. Considering that the conventional grinding method using standard grinding wheels and the kinematic capabilities of the tool grinding machine was used to create form tools, it can be assumed that the grinding speeds, the type of grain size, the grain density, and the geometry of each grinding wheel caused these differences. On the other hand, the grinding strategies and their use from the side or the periphery of the grinding wheel in contact with the form tool are influential in this field.

Compared to the grinding method, more uniformity can be seen on the flank surface of the form tools made using the electro-discharge wire-cutting method. Although the number of passes plays a role in the fineness and coarseness of the resulting surface texture and the thickness of the recast layer, the tendency toward a decrease or increase in the mentioned characteristics can be simultaneously seen in all areas of the free surface of the form tool. On the other hand, the contact area of the wire with different areas of form contour is directly related to their concavity, convexity, and radius. However, these effects are more tangible to consider in comparison with the qualitative differences of the grinding method.

**Figure 19:**
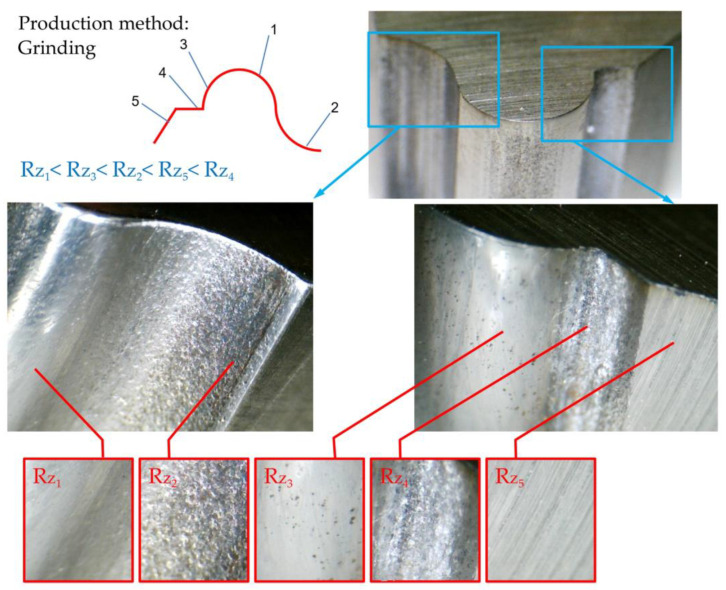
The surface finishing of form tools made using the grinding method.

According to the access of each area of the form tool to the grinding wheel and which area of the side or periphery of the grinding wheel was in contact with that area at which cutting speed, different roughnesses were created. In Figure 18, five areas of the flank surface of the form tool made through grinding are named, roughness measurements are performed for each point, and finally, they are compared. As shown in Figure 19, the roughness of the five named areas was equal to them and had a relatively identical decrease or increase concerning the number of passes. In this way, the flank surface roughness was measured for the form tools made with a wire-cutting machine.

**Figure 20:**
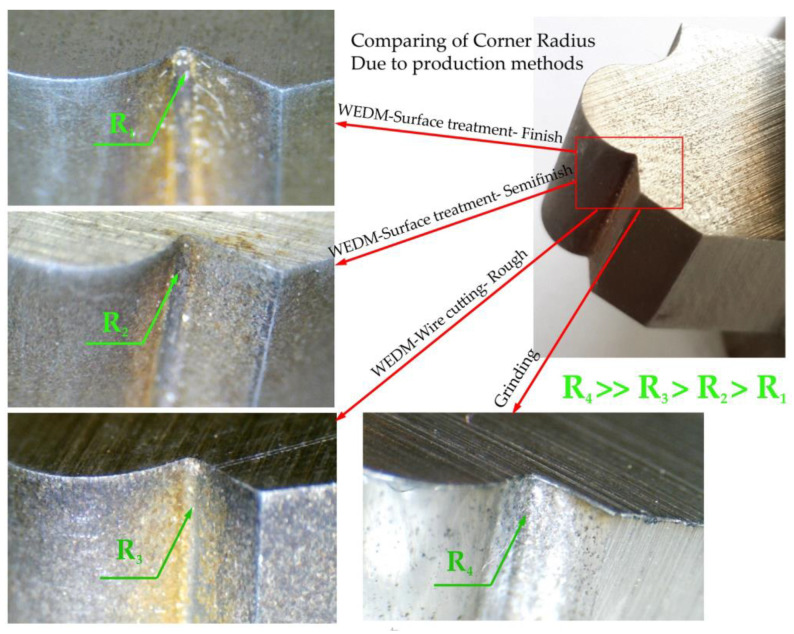
Comparison of the ability to manufacture an inner corner using the method of grinding and wire electro discharge machining (WEDM) (in three states: roughing, semi-finishing, and finishing).

For four form tools made using different methods, the inner corner of the form contour, which is the meeting point of the convex curved surface and the flat surface, was investigated as one of the critical points. Considering that the diameter of the used wire was 0.25 mm, the difference between the inner corner’s radius and the wire’s radius was used as a basis. As shown in Figure 20, the minor corner radius was for the form tool made using the three-pass electrical discharge method. The tools made with two and one passes were in the following ranks, and with a vast difference, the tools made by the grinding method were located. This can be a factor in choosing the method of manufacturing the form tool according to the geometry of the form contour. Especially in the case of internal features and small concavities, such as internal corners, narrow grooves, and sharp internal angles, there are limitations to the grinding method.

One thing that plays a role in chip formation, flow, and self-breakability is the texture direction of the rake surface of the form tool. In previous research, some researchers have studied the orientation of the existing machining texture on the tool’s rake face, which was related to the manufacturing stage of the cutting tool. When measuring the volume of material adhering to the rake face of the cutting tools (after machining), it was found that the highest stickiness occurred in the case where the direction was parallel to the in-feed direction of the cutting tool. According to recent explanations, because the production method of HSS blades (using a vertical cup surface grinder) was such that different orientations of the surface texture were created on the rake surface, it is possible that there were differences in the results of the tests, which were the source of the error. Regardless of this, in this research, this effect was ignored.

## 4. Conclusions

In the present study, a comparison between two methods of grinding and wire-cutting electro-discharge machining was carried out. To compare different production methods, a multi-functional form tool with different cutting-edge shapes was designed and, using a grinding machine, a five-axis wire cut machine was made. The form tools created using the wire-cutting method were made with three different machining states: rough, semi-finished, and finished. The chip formation of each form tool was studied through a set of designed experiments. The machining forces were recorded during form turning, and the results showed that the grinding method and WEDM with three passes had somewhat the same outcomes and could be good choices for form cutting in terms of surface finishing and a reduction in machining forces. The results of the experimental test showed that the chip formation of the finished surface of the wire cut tool was close to the ground tools. Moreover, the life of tools in wear creation was compared and showed that the tool produced by the three passes machining of the wire cutting method had better performance than other techniques. In addition, the manufacturing method of form tools can affect the final product precision. The WEDM product with a finishing surface (three passes machining) had the best performance by which to produce more specific products. The minimum recast layer on the flank surface of the form tools was the reason for the highest precision of this tool. Furthermore, the wire-cutting method with high surface finishing gave the best results for the production of form tools with complex profiles. This research is valuable for researchers seeking to advance methods in the fabrication of form-cutting tools, especially by adapting the methodologies discussed in recent articles. The development of manufacturing methods for new materials of cutting tools can be considered and the insights gained from this research can be utilized for the analysis of chip formation in form-turning operations. By incorporating discussions related to manufacturing costs for each method, it is possible to examine both technical and economic criteria in the production of form-cutting tools.

## Figures and Tables

**Table 1:** The chemical composition and material properties of the utilized HSS.

C	Cr	W	Mo	V	Co	Hardness (HRC)	Density (kg/m^3^)	Young’s Modulus (GPa)	Ultimate Strength (MPa)
1.1	3.9	1.6	9.5	1.2	8.25	67	7850	207	2560

**Table 2:** Machining conditions according to effective machining parameters.

(Cutting Tool Production Methods)	(Grinding)	(Wire Cutting Three Passes—Finish)	(Wire Cutting Two Passes—Semi Finish)	(Wire Cutting One Pass—Rough)
(Machining Condition)				
(Roughing turning) Spindle Speed 1 (rev/min): 125	DRG	DRW3	DRW2	DRW1
Feed per revolution 1 (mm/rev): 0.2				
(Semi-finishing turning) Spindle Speed 2 (rev/min): 180	DSG	DSW3	DSW2	DSW1
Feed per revolution 2 (mm/rev): 0.14				
(Finishing turning) Spindle Speed 3 (rev/min): 250	DFG	DFW3	DFW2	DFW1
Feed per revolution 3 (mm/rev): 0.08				

**Table 3:** Machining forces of the form-turning process based on different manufacturing methods.

Production Method	F_t_ (kgf)		F_r_ (kgf)		F_shear_ (kgf)	
	Mean Load	Peak Load	Mean Load	Peak Load	Mean Load	Peak Load
DW1	165	246	1578	2645	−1598	3514
DW2	154	221	1211	2450	−1149	1865
DW3	105	185	605	2028	248	2238
DFG	103	183	654	1853	552	2423

## Data Availability

No datasets were generated or analyzed during the current study.

## Glossary

AISI	American Iron and Steel Institute
CAD	Computer-Aided Design
CAM	Computer-Aided Manufacturing
CBN	Cubic Boron Nitride
CNC	Computer Numerical Control
DFG	Dry Machining, Finishing Operation, Manufacturing by Grinding Process
DOE	Design of Experiments
DPG	Dry Machining, Roughing Operation, Manufacturing by Grinding Process
DRW1	Dry Machining, Roughing Operation, Manufacturing by WEDM-1 Passes
DRW2	Dry Machining, Roughing Operation, Manufacturing by WEDM-2 Passes
DRW3	Dry Machining, Roughing Operation, Manufacturing by WEDM-3 Passes
DSW1	Dry Machining, Semi-Finishing Operation, Manufacturing by WEDM-1 Passes
DSW2	Dry Machining, Semi-Finishing Operation, Manufacturing by WEDM-2 Passes
DSW3	Dry Machining, Semi-Finishing Operation, Manufacturing by WEDM-3 Passes
DFW1	Dry Machining, Finishing Operation, Manufacturing by WEDM-1 Passes
DFW2	Dry Machining, Finishing Operation, Manufacturing by WEDM-2 Passes
DFW3	Dry Machining, Finishing Operation, Manufacturing by WEDM-3 Passes
EDM	Electro Discharge Machining
EDS	Electro Discharge Sawing
HSS	High-Speed Steel
rpm	Revolutions Per Minute
WEDM	Wire Electrical Discharge Machining
